# Monoclonal antibodies for chronic migraine and medication overuse headache: A real-world study

**DOI:** 10.3389/fneur.2023.1129439

**Published:** 2023-03-03

**Authors:** Abouch V. Krymchantowski, Carla Jevoux, Ana Gabriela Krymchantowski, Raimundo Pereira Silva-Néto

**Affiliations:** ^1^Department of Neurology, Headache Center of Rio, Rio de Janeiro, Brazil; ^2^Department of Neurology, Federal University of the Parnaíba Delta, Parnaíba, Brazil

**Keywords:** chronic migraine, medication-overuse headache, monoclonal antibody, erenumab, galcanezumab, fremanezumab

## Abstract

**Background:**

Medication-overuse headache is highly prevalent in tertiary care centers. It may be a cause or consequence of the overuse of symptomatic medications for migraine attacks.

**Objective:**

We aimed to compare the efficacy of anti-CGRP monoclonal antibodies (mAbs) added to conventional pharmacological treatments in patients with chronic migraine (CM) and medication overuse headache (MOH).

**Methods:**

A cross-sectional, prospective, randomized, open study with real-world comparison groups of patients was carried out. The sample consisted of 200 patients with CM and MOH, who received the same approach to withdraw overused medications, started preventative treatment, and either did or did not receive mAbs.

**Results:**

A total of 172 patients (126 women and 46 men) were included in the study and divided into two groups: group one consisting of 58 patients (control) and group two of 114 patients who used mAbs added to conventional pharmacological agents. The mean age was 44.1 ± 13.6 years, ranging from 18 to 78 years. In the 3 months follow-up after starting the treatment, both groups presented headache frequency reduction, but those with monoclonal antibodies had a significantly higher reduction in the number of headache days and symptomatic medication intake when compared to the control (*p* < 0.0001).

**Conclusions:**

The addition of an anti-CGRP monoclonal antibody to the treatment for medication overuse headaches in chronic migraineurs may result in decreasing headache frequency and symptomatic medication use when compared to conventional treatments with drugs.

## Introduction

Medication overuse headache (MOH) is a secondary headache associated with the overuse of symptomatic medications (SM) for the acute treatment of migraine (1, 2). It is a highly prevalent and debilitating neurological condition, especially in tertiary centers (3–5). Its prevalence in the general population is also high, reaching 1%-7% of the world population (1).

MOH is characterized by the presence of ≥15 days/month of headache in patients with preexisting migraine or tension-type headache on SM overuse for at least 3 months. Medication overuse is defined as consuming simple analgesics or non-steroidal anti-inflammatory drugs for 15 or more days per month or using a combination of analgesics and caffeine, triptans, and/or ergotamine derivatives for 10 or more days per month. For opioids, benzodiazepines, and barbiturates, the criteria are stricter, with the possibility of induction or transformation into MOH, when ingestion occurs on two or more days of the week (2).

The management of MOH consists of the sudden withdrawal of overused medications and the initiation of preventive treatment (1, 6–10). This should be the best approach and has been long the chosen treatment strategy (1, 9, 11, 12). Sometimes treatment may require bridging transition therapies such as dihydroergotamine or prednisone during the first few days of detoxification due to escalating headache and withdrawal symptoms (1, 9, 11, 13).

Recent clinical trials have shown that anti-CGRP mAbs are effective in interrupting the pattern of drug overuse and improving MOH (14).

The aim of this study was to compare the outcomes of chronic migraineurs with medication overuse headaches treated with and without an anti-CGRP monoclonal antibody added to an established treatment using education, drug overuse interruption, and initiation of preventive medication.

## Methods

### Study design and patients

A cross-sectional, prospective, and randomized study was carried out. The sample consisted of 200 patients with chronic migraines and medication overuse headaches treated at a tertiary clinic. Data were collected from September 2020 to May 2022 at a single headache center. The study protocol was approved by the institutional review board and all participants gave written informed consent to participate in this study.

### Inclusion and exclusion criteria

Consecutive new patients older than 18 years diagnosed with chronic migraine and MOH according to ICHD-3 criteria (2) and who agreed to participate were included in this study. To all patients, the nature of MOH and migraine was clearly presented. In addition, the fundamentals of the treatment, reasons for withdrawal, advantages of initiating prevention, and the potential usefulness of the anti-CGRP monoclonal antibodies were emphatically illustrated. The subjects who used onabotulinumtoxinA in the last 6 months or who were using daily preventive medications and had dose adjustments in the last 2 months were excluded from the study. Moreover, women who were pregnant or who were planning to initiate a pregnancy within the next 12 months were also excluded.

### Data collection

During a long-lasting initial consultation (1:00–1:15 h), all patients were thoroughly evaluated and informed about MOH. All received a headache diary to be filled out, which included headache features and detailed information regarding the use of SM. This diary had to be presented at 3 and 6 months to the respective physicians.

The patients were also instructed, verbally and with written material, to perform a sudden discontinuation of the currently used symptomatic drugs. A 5-day bridge treatment was started with prednisone (60 mg/day for 3 days, 40 mg/day for 1 day, and 20 mg/day during one more day) or indomethacin (50 mg twice a day), and treatment with traditional preventive medications, including either beta-blockers, tricyclic antidepressants, calcium channel blockers, and/or anticonvulsants (monotherapy or polytherapy) was initiated from the 6th day onwards (Table 1). Additionally, all received a prescription of triptan plus an anti-inflammatory agent, which could be used at a maximum frequency of 2 days per week.

**Table 1:** Conventional pharmacological treatment used in 172 patients with chronic migraine and medication overuse headache.

**Migraine prophylactic drugs**	**Patients (n; %)**
Topiramate and nortriptyline	86 (50.0)
Divalproex sodium extended-release	51 (29.7)
Atenolol and nortriptyline	25 (14.5)
Atenolol, nortriptyline and flunarizine	10 (5.8)

During the first consultation, the entire sample of patients was randomized into two groups on a 1:2 sequence. Group one received the treatment described above and group two received the exact same approach plus the information regarding the new therapy for migraine and its potential efficacy in further reducing headache frequency and SM overuse. In addition, group two received a prescription for one of the three mAbs available in Brazil, 70 mg of erenumab once a month, 240 mg (loading dose), followed by 120 mg, of galcanezumab once a month, or 225 mg of fremanezumab once a month, with the clear instruction to use the one more easily or less expensively acquired. There was an emphasis, presented to the patients, on their similarity in efficacy and tolerability. This strategy was employed since mAbs in Brazil must be purchased by the patients themselves and are not provided by the public health system or by most health insurance plans.

The choice of traditional migraine prophylactics was based on the experience of the treating physicians as well as the existence of comorbidities. At four weeks, all the patients had to inform their physician of their choice for a specific mAb or the choice for not using a mAb initially.

Based on this approach, and at the first follow-up visit after 3 months, group one followed the treatment strategies, and group two, in addition to the treatment, used one of the three mAbs. This first group was the control group. Twenty-eight patients of the whole sample were unavailable during the follow-up.

The study sample consisted, therefore, of 172 patients who returned for evaluation after having followed the instructions. They were distributed to 58 patients in group one and 114 in group two (27 patients with erenumab, 40 patients who used galcanezumab, and 47 patients who were treated with fremanezumab). Patients were followed for a period of 6 months to assess the treatment outcomes.

### Statistical analysis

The Statistical Package for the Social Sciences (SPSS^®^) version 18.2.2 was used for statistical analysis. Quantitative variables were expressed as mean, standard deviation, and minimum and maximum values, while qualitative variables were expressed as absolute and relative frequencies. The chi-square test with the Yates correction and Students' *t*-test were used for the difference in means of unpaired samples.

## Results

A total of 172 patients (126 women and 46 men) were included in the study and divided into two groups: group one consisting of 58 patients (control) and group two of 114 patients who used mAbs (27 patients used erenumab, 40 patients used galcanezumab, and 47 patients used fremanezumab). The mean age was 44.1 ± 13.6 years, ranging from 18 to 78 years. All patients had a headache for ≥15 days a month, for at least 3 months, and had medication-overuse according to the IHCD-3 (2). The common complaints of functional incapacity for work and quality life impairment were referred to by most of the patients from the two groups. However, there was an absence, in this study, of numbers and scales to compare patient samples in this way. The patients received one or more prophylactic agents for migraine prevention, which included beta-blockers, tricyclic antidepressants, calcium channel blockers, and/or neuromodulators (monotherapy or polytherapy). Before the treatment approaches, all patients were overusing various SM, such as common analgesics, non-steroidal anti-inflammatory drugs, triptans, ergots, and benzodiazepines for ≥20 days per month. No differences were revealed between the groups regarding the overuse of specific classes of symptomatic medications. The characteristics of each group regarding age at diagnosis, age of pain onset, and latency to diagnosis are shown in the Table 2.

**Table 2:** Distribution of sex, age of pain onset and latency until diagnosis of 172 patients with chronic migraine and medication overuse headache.

**Variables**	**Comparison groups**		***p value***
	**Group 1 (Control) (*n* = 58)**	**Group 2 (use of mAbs) (*n* = 114)**	
Sex			0.997
Female (*n*; %)	42 (72.4)	84 (73.7)	
Male (*n*; %)	16 (27.6)	30 (26.3)	
Age at diagnosis (years)			0.683
Average (SD)	42.6 ± 13.3	44.8 ± 13.8	
Variation	19–65	18–78	
Age of onset of pain (years)			0.260
Average (SD)	19.7 ± 11.6	16.9 ± 6.8	
Variation	8–54	5–39	
Latency until diagnosis (years)			0.372
Average (SD)	22.8 ± 12.9	28.0 ± 14.0	
Variation	3–50	6–61	

Table 3 shows that before treatment, all patients who were distributed in the two groups had 15 or more headache days per month in the last 3 months. The mean headache frequency in groups one and two was, respectively, 22.3 ± 7.1 and 24.0 ± 4.6 days per month. In the 3 months of follow-up with prophylactic treatment, there was a reduction in the number of headache days in both groups, predominantly in the group treated with mAbs (*p* < 0.0001).

**Table 3:** Number of headache days before and after starting monoclonal antibodies.

**Variables**	**Comparison groups**		***p value***
	**Group 1 (Control)**	**Group 2 (use of mAbs)**	
Before starting mAbs (in the last three months) (*n*; SD)	24.0 ± 4.6	22.3 ± 7.1	0.346
After starting mAbs (from the 1st to the 3th month) (*n*; SD)	12.7 ± 8.2	8.4 ± 6.4	< 0.0001

SD, standard deviation; mAbs, monoclonal antibodies; *p value calculated using the student's *t* test.

Table 4 shows the overuse of SM in both groups during the previous 3 months before treatment. The mean frequency of analgesic intake days in the two groups was, respectively, 26.9 ± 3.2 and 28.3 ± 2.4 days per month. After 3 months of prophylactic treatment, there was a reduction in the number of days of analgesic intake in both groups, predominantly in the group treated with mAbs (*p* < 0.0001).

**Table 4:** Number of days per month of symptomatic medications use before and after treatment with monoclonal antibodies.

**Variables**	**Comparison groups**		***p value***
	**Group 1 (Control)**	**Group 2 (use of mAbs)**	
At baseline (*n*; SD)	28.3 ± 2.4	26.9 ± 3.2	0.158
After three months of treatment (*n*; SD)	12.2 ± 6.7	5.9 ± 3.3	< 0.0001

SD, standard deviation; SM, symptomatic medications; mAbs, monoclonal antibodies; *p value calculated using the student's *t* test.

Adverse effects of mAbs occurred only in patients who used erenumab (constipation) and galcanezumab (injection site pain, nasopharyngitis, non-specific malaise, and vertigo), as shown in Table 5.

**Table 5:** Adverse effects of mAbs in 114 patients with chronic migraine and medication overuse headache.

**Side effects**	***n*; %**	**Monoclonal antibodies**		
		**Erenumab (*n* = 27)**	**Galcanezumab (*n* = 40)**	**Fremanezumab (*n* = 47)**
Intestinal constipation	6 (4.9)	6	0	0
Pain at the injection site	3 (2.4)	0	3	0
Nasopharyngitis	1 (0.8)	0	1	0
Non-specific malaise	1 (0.8)	0	1	0
Vertigo	1 (0.8)	0	1	0

## Discussion

The efficacy of anti-CGRP mAbs for chronic migraine and medication-overuse headaches has been demonstrated in various subgroup analyses (15–17). Initially, specific subpopulations of patients with chronic migraine and MOH (15–17) were studied in the pivotal studies with the mAbs, but recently real-world studies aimed at these subsets of patients were also carried out and corroborated the potential usefulness of the mAbs, even in subjects who did not stop using the overused medications (14, 18, 19).

However, despite the similarity with other clinical trials using monoclonal antibodies for chronic migraineurs and medication overuse headaches (14, 18, 19) in our study, the two groups of patients were equally instructed about the potential headache progression and chronification caused by the excessive use of SM (7, 9, 10, 12, 20).

Therefore, one might argue whether the use of anti-CGRP mAbs would add value to the improvement figures, with the supposed absence of differences in outcomes between the groups. Additionally, our study included only subjects with chronic migraine and MOH, different from other samples in which MOH was diagnosed in just some of the chronic migraineurs (14, 18, 19). This study is the first Brazilian study on MOH and the comparative use of mAbs and traditional pharmacological approaches.

Withdrawing overused medications, providing information, and, for some populations of patients, starting prevention immediately are the most effective treatments for medication overuse headache (12, 20, 21). The question of whether adding a mAb could result in better outcomes was our main concern when planning the present study, despite the existence of emerging data on efficacy from trials with anti-CGRP mAbs without acute medication withdrawal (22). In addition, no comparative trials using mAbs for MOH patients were found for comparisons. One might argue whether funding restrictions could explain such a gap but we decided to conduct the present investigation using the patient's own resources to decide how each monoclonal antibody would be chosen. Moreover, since the most effective treatment for MOH is to educate the patient, withdraw SM, and start preventative treatment, we aimed at investigating whether the addition of an anti-CGRP mAb could result in better outcomes. Nevertheless, despite the small sample size, open design, and bias in the preventive pharmacological agents chosen for the various patients, we were able to observe differences in outcomes between the group who received mAbs and the group who only used the traditional approach.

A recent study from Italy included patients with MOH who underwent in-hospital sudden detoxification and compared them with a sample who did not perform detoxification, despite being advised to withdraw according to local recommendations (19). Although the patients enrolled were a small subset of a total of 401 patients, and only 25% (*n* = 28) went through the in-patient procedures of detoxification in comparison to 75% who did not (*n* = 83), the study design was interesting and evaluated whether starting a mAb during the 1st week of treatment (the last day of detoxification for those who did it) would result in different outcomes. The study did not clearly demonstrate the patterns of rescue medications used during the withdrawal phase or whether steroids were used daily but enrolled patients overusing symptomatic medications for ≥28 days/month (19). There were no differences between the two groups of overused classes of pharmacological agents as well as in demographics or baseline headache features and frequencies. The number of previous failures of migraine treatments, including onabotulinumtoxinA, was also similar among studied patients. After 3 months of treatment with either erenumab or galcanezumab, the headache was reduced in 47 out of 83 (57%) of the patients who did not detoxify vs. 18 out of 28 (64%) of the group who withdrew; (*p* = 0.4788). Monthly headache days (MHDs) significantly reduced from 29.93 ± 0.35 to 18.63 ± 9.32 (*p* < 0.0001) by the 3rd month of treatment as well.

Regarding outcomes related to quality of life, the HIT-6 score significantly improved by the 3rd month compared to the baseline (65.99 ± 9.21 vs. 58.57 ± 7.65; *p* < 0.0001), and 57 out of 111 (51%) patients achieved ≥50% reduction in monthly headache days, equally distributed between patients who underwent in-patient withdrawal and the ones who did not (*p* = 0.839). The authors concluded that the early initiation of an anti-CGRP mAb may be effective in the treatment of patients with MOH irrespective of detoxification but did not compare the outcomes observed with specific monoclonal antibodies (19).

In another real-world study from Italy, the use of erenumab converted 68% of 91 chronic migraineurs to an episodic pattern and reduced the overuse of SM to a non-overuse pattern within 6 months (23). Galcanezumab has also been shown to be effective in preventing migraine even in patients with previous failures with conventional treatments, but, in addition, it was able to promote a clinically significant decrease in the number of days of SM intake in the 1st month of treatment (24). However, these two studies did not include specific samples of patients diagnosed with MOH, did not compare different mAbs, nor did they include a control group that did not use any anti-CGRP mAbs.

Another study using *post-hoc* analyses was conducted to assess the efficacy of fremanezumab in patients with and without medication overuse. The change from baseline (28-day pretreatment period) in the monthly average number of migraine days during the 12-week treatment period, the monthly average number of days of any acute headache medication use, and the change in the monthly average number of headache days during the 12-week treatment period were the outcomes evaluated. In addition, patients with medication overuse at baseline were assessed for reversion to no medication overuse. Contrary to our study, this study did not include an entire population of patients with medication overuse, but rather, nearly 50% (587 out of 1,130) of chronic migraineurs were overusing SM (17, 25).

Interestingly, the three mAbs available in Brazil at the time of writing, and used in our study, were quite similar regarding headache frequency reduction, despite the fact that these comparisons were not the scope of this trial. In addition, the present trial demonstrated a very favorable profile of tolerability and adhesion with similar dropout rates between groups. Regarding adverse events, the reported figures were mild in presentation. One might speculate that once the patients were able to acquire the expensive mAbs, they adhered to the treatment strategy, regardless of other variables, which resulted in positive outcomes. In addition, it is possible that some of the patients who were not available for follow-up in both groups did so due to financial issues, since consultations and medications were carried out under the patient's auspices. On the other hand, part of the high adhesion rates may also have been determined by the unprecedented comprehensive approach, the premise of the present trial, accomplished in the tertiary centers.

This is the first real-world study that compared the use of an anti-CGRP mAb with traditional treatments in patients with MOH including a control group. We highlight as merits of the study the fact that all patients were informed about the nature of MOH and migraine, the rationale for treatment, reasons for withdrawing SM, the advantages of initiating prevention, and the potential usefulness of the mAb anti-CGRP.

Limitations of this study include a single center as the study site and relatively small sample size. The study was open, not allowing definitive conclusions about the treatments. There were methodological limitations as well such as bridging treatment with different drugs, but with no difference between one and the other. The use of preventive drugs with different mechanisms of action based on the physician's clinical experience or bias, and the addition of three different mAbs did not represent confounding factors, but rather, facilitation for the patient's process of acquiring therapy. In addition, the therapeutic response of each one was quite similar. However, this was not a funded study, which would have allowed the inclusion of more subjects and a better design, but only evaluated real-world patients seeking treatment in a traditional tertiary center in Brazil. We speculate that the peculiar characteristics of chronic migraineurs and those with MOH in tertiary centers of Brazil may have powered the present study (9, 26). Most of the patients were highly motivated by the comprehensive approach, some undergoing treatment for the first time, and the main limitation of the follow-ups was the cost of the care and treatments (9, 26, 27). Despite that, we were surprised by the high adhesion to treatment even when the treatment had to be purchased or provided by the patients themselves. This could have been related to the high motivation and a previous history of consulting numerous physicians without ever receiving a diagnosis (26, 27).

## Conclusions

The treatment with mAbs added to traditional pharmacological agents in patients with chronic migraine and MOH leads to a higher reduction in the number of headache days and symptomatic medication use when compared to conventional treatment with drugs. Adherence to the treatment with a mAb was high, despite costs not being reimbursed by governments or insurance companies as observed in Brazil. Controlled studies are necessary to corroborate our observations.

## Data availability statement

The raw data supporting the conclusions of this article will be made available by the authors, without undue reservation.

## Ethics statement

The studies involving human participants were reviewed and approved by Federal University of Piauí. The patients/participants provided their written informed consent to participate in this study.

## Author contributions

AVK: conception and design, acquisition of data, analysis and interpretation of data, drafting the manuscript, and revising it for intellectual content. CJ: acquisition of data, analysis and interpretation of data, and drafting the manuscript. AGK: analysis and interpretation of data and drafting the manuscript. RS-N: conception and design, acquisition of data, analysis and interpretation of data, drafting the manuscript, and revising it for intellectual content. All authors approved the final version of the manuscript.
